# Supplementary material for: Causal Relationship between Aging and Anorexia Nervosa: A White-Matter-Microstructure-Mediated Mendelian Randomization Analysis
Source: Biomedicines. 2024 Aug 16;12(8):1874. doi: 10.3390/biomedicines12081874 (PMC11351342; doi:10.3390/biomedicines12081874)
Supplement: Supplementary file 1 [file biomedicines-12-01874-s001.zip › Supplementary Figure/Figure S4/Singe SNP plot of MR analysis for TL to ubm-b-1689.pdf]

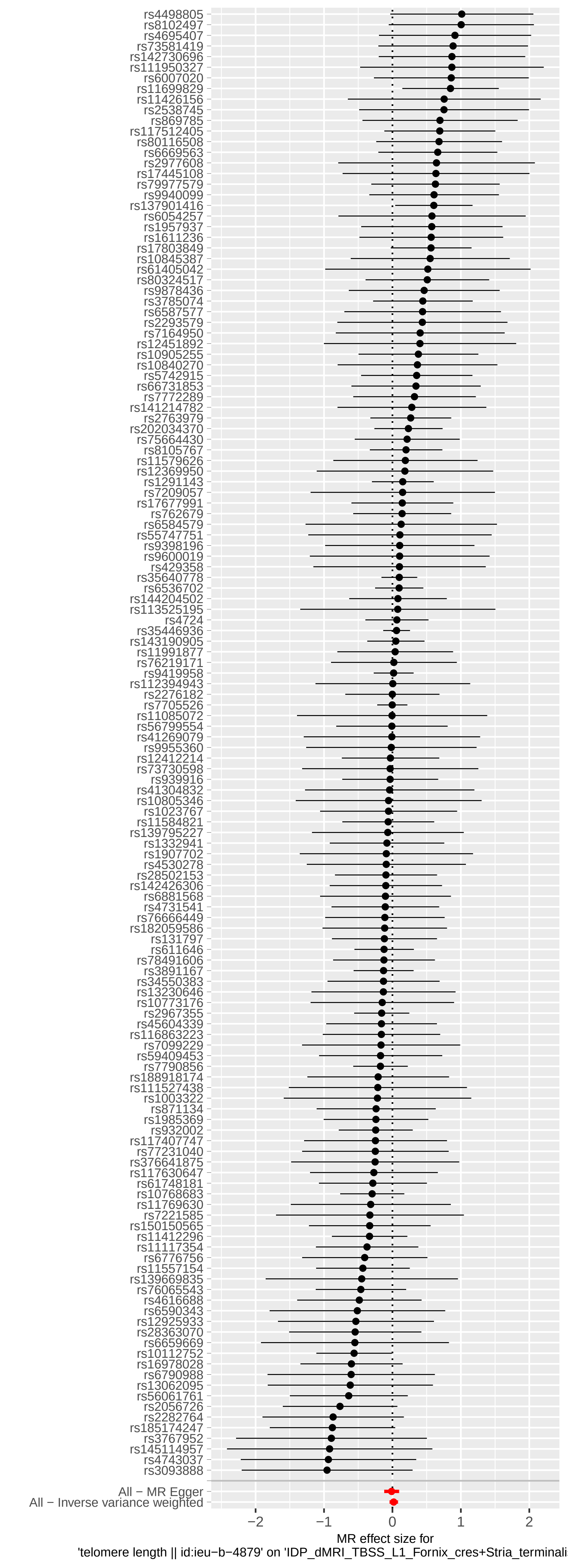
rs4498805
rs8102497
rs4695407
rs73581419
rs142730696
rs111950327
rs6007020
rs11699829
rs11426156
rs2538745
rs869785
rs117512405
rs80116508
rs6669563
rs2977608
rs17445108
rs79977579
rs9940099
rs137901416
rs6054257
rs1957937
rs1611236
rs17803849
rs10845387
rs61405042
rs80324517
rs9878436
rs3785074
rs6587577
rs2293579
rs7164950
rs12451892
rs10905255
rs10840270
rs5742915
rs66731853
rs7772289
rs141214782
rs2763979
rs202034370
rs75664430
rs8105767
rs11579626
rs12369950
rs1291143
rs7209057
rs17677991
rs762679
rs6584579
rs55747751
rs9398196
rs9600019
rs429358
rs35640778
rs6536702
rs144204502
rs113525195
rs4724
rs35446936
rs143190905
rs11991877
rs76219171
rs9419958
rs112394943
rs2276182
rs7705526
rs11085072
rs56799554
rs41269079
rs9955360
rs12412214
rs73730598
rs939916
rs41304832
rs10805346
rs1023767
rs11584821
rs139795227
rs1332941
rs1907702
rs4530278
rs28502153
rs142426306
rs6881568
rs4731541
rs76666449
rs182059586
rs131797
rs611646
rs78491606
rs3891167
rs34550383
rs13230646
rs10773176
rs2967355
rs45604339
rs116863223
rs7099229
rs59409453
rs7790856
rs188918174
rs111527438
rs1003322
rs871134
rs1985369
rs932002
rs117407747
rs77231040
rs376641875
rs117630647
rs61748181
rs10768683
rs11769630
rs7221585
rs150150565
rs11412296
rs11117354
rs6776756
rs11557154
rs139669835
rs76065543
rs4616688
rs6590343
rs12925933
rs28363070
rs6659669
rs10112752
rs16978028
rs6790988
rs13062095
rs56061761
rs2056726
rs2282764
rs185174247
rs3767952
rs145114957
rs4743037
rs3093888
All − MR Egger
All − Inverse variance weighted
−2
−1
0
1
2
MR effect size for
'telomere length || id:ieu−b−4879' on 'IDP_dMRI_TBSS_L1_Fornix_cres+Stria_terminali
